# Associations between Snacking and Weight Status among Adolescents 12–19 Years in the United States

**DOI:** 10.3390/nu11071486

**Published:** 2019-06-29

**Authors:** Gina L. Tripicchio, Alexandria Kachurak, Adam Davey, Regan L. Bailey, Lauren J. Dabritz, Jennifer O. Fisher

**Affiliations:** 1 Center for Obesity Research and Education, Temple University, Philadelphia, PA 19140, USA; 2 Behavioral Health and Nutrition, University of Delaware, Newark, DE 19716, USA; 3 Department of Nutrition Science, Purdue University, West Lafayette, IN 47907, USA

**Keywords:** snacking, obesity, energy-dense, nutrient poor foods and beverages, adolescents, behavioral health

## Abstract

Snacking is a significant contributor to energy intake among adolescents, but its association with weight status is unclear. To elucidate this association, data from 6545 adolescents (12–19 years) in the 2005–2016 National Health and Nutrition Examination Survey (NHANES) were analyzed. The mean number of daily snack occasions, mean snack size, and mean snack energy density were examined by weight classification (body mass index (BMI)-for-age percentiles: normal weight (NW) <85th; overweight (OW) ≥85th to <95th; obese (OB) ≥95th). Models included all snacking parameters, mean meal size, demographic characteristics, survey cycle year, and dietary reporting accuracy. Adolescents with NW consumed fewer snacks daily (1.69 (0.02) snacks/day) and smaller snacks per occasion (262.32 (4.41) calories (kcal)/snack) compared to adolescents with OW (1.85 (0.05) snacks/day, *p* = 0.005; 305.41 (8.84) kcal/snack, *p* < 0.001), and OB (1.97 (0.05) snacks/day; 339.60 (10.12) kcal/snack, both *p* < 0.001). Adolescents with OW and OB also consumed more added sugar, saturated fat and sodium from snacks, but had lower mean energy density per snack compared to snacks consumed by NW adolescents. US adolescents with OW and OB consume more snacks daily and more calories at each snacking occasion compared to adolescents with NW. Future studies should examine the prospective associations between snacking and weight status and impact on overall diet quality.

## 1. Introduction

Snacking (i.e., eating in between meals) has significantly increased in the United States (US) over the past 30 years alongside rates of pediatric obesity [1,2]. Adolescents consume approximately 20% of their daily energy intake as snacks, with salty and sweet foods contributing a majority of the snacking calories [3,4]. Current Dietary Guidelines for Americans recommend that adolescents consume nutrient-dense snacks to help them meet their nutritional needs [5]. However, whether snacking currently benefits dietary intake and growth among adolescents or promotes obesity is unknown [6,7]. 

Given that adolescents have the highest prevalence of obesity among children, understanding the relationship between snacking and weight status in this population is critically important [2]. Adolescents with obesity are at increased risk of diet-related co-morbidities including hypertension, type 2 diabetes, and dyslipidemia as well as psychosocial consequences such as stress, stigma and depression [8,9]. Compared to other pediatric populations, adolescents with obesity are also more likely to remain obese as adults, increasing their risk for additional morbidity and mortality [10,11]. A myriad of foods have been targeted as key dietary drivers of obesity, including sugary drinks, fast foods, and energy-dense foods, which are often consumed as snacks [12]. These foods can contribute “empty” and excess calories that do not provide essential nutrients to help adolescents meet their nutritional needs. For these reasons, snacks are often targeted to achieve caloric reduction in obesity treatment programs for pediatric populations [13,14]. However, a better understanding of snacking behaviors, including optimal frequency, size, and types of snacking for healthy weight are unknown [15]. Given the complexities involved in measuring and defining snacking, the literature is currently mixed on whether snacking has a positive or negative impact on overall nutrient intake and body composition [16]. Even less is known about the effects in children. As such, a comprehensive conceptualization of snacking is needed to develop salient intervention targets to quell the current trends in obesity and related diseases in adolescents. 

Studies examining the association between snacking and weight status in adolescents and across other age groups have found no association or a protective effect of snacking on obesity risk [17,18]. However, many studies rely on parent-reports of adolescent intake, examine snacking in the context of other behaviors (e.g., snacking while watching television), or look at associations with specific snacking patterns (e.g., high-fat, high-sugar, high-energy density snack foods). Variability in the definitions of snacking as well as a failure to account for potential bias in reporting dietary intake limit the ability to estimate true associations between snacking and weight outcomes [19,20]. Much of the work to date has combined overweight and obesity classifications, which precludes the ability to examine independent associations and can mask trends that are contributing to rising rates of obesity among adolescents [19,21]. Finally, many studies have focused on the frequency of snacking at the exclusion of other potentially important dimensions, like snack size [21]. A number of studies have looked at eating frequency and found decreased risk of obesity, metabolic disease and cardiovascular outcomes [22,23]. However, these studies, which combine all eating occasions into a single outcome, might be masking the independent associations of snack intake on weight status. In order to address these limitations, this study considered multiple parameters of snacks consumed by adolescents, accounted for reporting bias of energy intake, and examined the associations using categorical weight classifications. 

Using a nationally representative sample of adolescents, the primary aim of this research was to evaluate the association of weight classification with snack frequency (number of snacking occasions per day), snack size (calories (kcal) per snacking occasions), and snack energy density (calories per gram (g) per snack occasion). Secondary aims included examining nutrients (added sugar, saturated fats, sodium) from snacking by weight classification, given their association with poor diet quality and disease risk [5]. Evidence of the associations between snacking and weight status will address current gaps in the literature and provide evidence to inform behavioral approaches and recommendations regarding snacking and weight status in US adolescents. 

## 2. Materials and Methods 

Data from 6545 adolescents, aged 12–19 years, who participated in the 2005–2016 National Health and Nutrition Examination Surveys (NHANES) were included in this analysis. NHANES is an ongoing, nationally representative study of the nutritional and health status of the civilian, non-institutionalized US population. NHANES uses a complex, multistage, probability sampling design with county as the primary sampling unit from which clusters of households and participants are selected. Data are collected by trained study staff and participants complete an in-home interview where demographic and health-related information are collected, followed by a medical examination and a 24-h recall [24]. A second 24-h recall is collected via phone. Additional details about the NHANES design and data collection procedures are available elsewhere [25]. 

### 2.1. Measures 

#### 2.1.1. Demographics 

Age (years), gender (male/female), and race/ethnicity (non-Hispanic white, Hispanic/Mexican American, non-Hispanic black, other) were collected for the participating adolescents from the in-home interview. Head of household (HH) age, marital status, education level, and income were also collected. The ratio of income to poverty (RIP) was estimated using family income relative to the poverty threshold; families greater than 125% of the poverty threshold were classified as high RIP and those less than 125% of the poverty threshold were classified as low RIP [26]. 

#### 2.1.2. Weight Status 

Adolescent height and weight were objectively measured by trained staff in the mobile examination center [27]. Height was measured to the nearest 0.1 cm using a stadiometer and weight was measured to the nearest 0.1 kg using a digital scale. Height and weight were used to calculate body mass index (BMI) and date of birth and gender were used to derive age-and-sex specific BMI-percentiles (BMI%) using the CDC growth charts [28]. Weight status was classified according to the following BMI% ranges: normal weight (NW) <85th; overweight (OW) ≥85th to <95th; and obesity (OB) ≥95th [29]. 

#### 2.1.3. Dietary Intake 

Dietary data were collected via two 24-h dietary recalls [30]. The first recall was conducted in person and the second occurred via phone. Adolescents (12 years and older) self-reported their dietary intake using the United States Department of Agriculture (USDA) Automated Multiple-Pass method [31,32]. Eating occasions were characterized by participants using a pre-determined list (e.g., “breakfast”). Snacking occasions were those identified by participants as “snacks.” Additionally, given the general definition of snacking that refers to eating in between meals, those occasions labeled as “beverages” or “extended consumption” were also included. Only adolescents with two days of dietary data were included in the analyses (1720 participants were excluded due to having only one dietary recall). The mean of two 24-h dietary recalls was used to estimate mean daily snacking occasions (number of daily snacking occasions), mean snack size (kcal/occasion), and mean snack energy density (kcal/g/occasion). Dietary weights were used to adjust for dietary nonresponse and day of the week [30]. Based on work done in a previous study by this team, initial models were evaluated with and without snacks containing trivial energy (eating occasions <5 kcal) [20]. Results were similar, therefore snack occasions with <5 kcal were excluded.

The Food Patterns Equivalents Database (FPED) was used to convert the What We Eat in America (WWEIA) dietary data to the USDA Food Pattern components [33]. Teaspoons of added sugar were converted to grams by multiplying by 4.2 and then grams were converted to calories by multiplying by 4; grams of saturated fat were converted to calories by multiplying by 9; sodium was reported in milligrams (mg).

### 2.2. Dietary Reporting Accuracy

The ratio of reported energy intake to estimated energy requirements (EI/EER) was used to assess dietary reporting accuracy. Estimated energy requirements were calculated using Dietary Reference Intake equations based on age, gender, weight, and physical activity level [34]. A “low active” level of physical activity (≥1.4 to <1.6) was assumed for all adolescents [35]. Following the methods in Murakami and Livingstone, the EI/EER ratio was used as a covariate in the analyses to account for EI reporting error (rather than removing implausible cases) so as not to bias sample selection [19].

### 2.3. Analyses

Descriptive statistics were generated and presented as means (standard errors) for continuous variables and percentages for categorical variables. Multiple linear regression models evaluated weight status as a predictor of each snacking parameter, adjusting for all other snacking parameters along with mean meal size (to account for non-snacking intake), survey cycle year and estimated energy reporting accuracy. Poisson regression using backward elimination was used to examine potential covariates. Child gender, age, race/ethnicity origin, RIP, survey cycle year, and HH age were retained and included as covariates in all models. Multiple linear regression models also examined the extent to which added sugar, saturated fats, sodium consumed from snacks varied by weight status. The primary outcome models examined total snacks including both foods and beverages. Supplementary models examined snacks from foods only and beverages only, separately. Although the central limit theorem posits that minor violations of normality of the dependent variables would not affect study conclusions, all analyses were repeated using 1000 bootstrapped replications to verify this; substantive conclusions were identical. All analyses were conducted in Stata (version 15.1; StataCorp, College Station, TX, USA).

## 3. Results

### 3.1. Demographics

Participants (*n* = 6545) were 15.4 (0.05) years, 57.7% non-Hispanic white, 20.3% Mexican American/Hispanic, 14.7% non-Hispanic black, and 50.3% female (Table 1). Examining adolescent weight status, 64.6% were classified as normal weight, 15.3% were overweight, and 20.1% had obesity. Snacks were consumed on average 1.77 (0.02) occasions/day, were 284.54 (4.60) kcal/occasion, and had an energy density of 2.56 (0.02) kcal/g/occasion. The HH mean age was 43.9 (0.36) years, a majority (72.6%) reported being married or partnered, and 58.2% reported education beyond a high school diploma.

### 3.2. Snacking Characteristics by Weight Classification

Adolescents with NW had an average of 1.69 (0.02) snacking occasions/day compared to adolescents with OW (1.85 (0.05) occasions/day, *p* = 0.005) and adolescents with OB (1.97 (0.05) occasions/day, *p* < 0.001) (Table 2). Mean snack size was 262.32 (4.41) kcal/occasion for adolescents with NW, 305.41 (8.84) kcal/occasion for adolescents with OW (*p* < 0.001) and 339.60 (10.12) kcal/occasion for adolescents with OB (*p* < 0.001). Mean snack energy density was 2.62 (0.03) kcal/g/occasion for adolescents with NW, 2.51 (0.06) kcal/g/occasion for adolescents with OW (*p* = 0.09), and 2.41 (0.06) kcal/g/occasion for adolescents with OB (*p* = 0.005). Total daily added sugar from all snacking occasions was 120.52 (3.78) kcal for adolescents with NW, 158.99 (8.12) kcal for adolescents with OW (*p* < 0.001) and 183.54 (9.50) kcal for adolescents with OB (*p* < 0.001) (Table 3). Total daily saturated fat from snacking occasions was 48.42 (1.40) kcal for adolescents with NW, 61.79 (2.65) kcal for adolescents with OW (*p* < 0.001), and 72.38 (4.31) kcal for adolescents with OB (*p* < 0.001). Totally daily sodium from snacking occasions was 456.04 (14.10) milligrams (mg)/day for adolescents with NW, 555.12 (25.02) mg/day for adolescents with OW (*p* = 0.002), and 677.37 (39.93) mg/day for adolescents with OB (*p* < 0.001). Snacking parameters also varied by gender and race/ethnicity; males consumed more frequent (1.96 (0.03)) occasions/day) and larger snacks (342.39 (7.60) kcal/occasion) compared to females (1.59 (0.03) occasions/day, *p* < 0.001; 227.28 (4.67) kcal/occasion, *p* < 0.001) and black adolescents consumed more frequent (1.91 (0.04) occasions/day), larger (319.78 (6.11) kcal/occasion), and more energy dense snacks (2.70 (0.04) kcal/g/occasion) compared to white adolescents (1.75 (0.03) occasions/day, *p* < 0.001; 285.66 (6.08) kcal/occasion, *p* < 0.001; 2.54 (0.04) kcal/g/occasion, *p* < 0.001).

## 4. Discussion

These nationally representative findings provide new evidence of the associations between snacking behaviors and weight status among US adolescents. The findings indicate that adolescents with OW and OB consume more snacks daily and more calories at each snacking occasion compared to adolescents who are classified as NW. Adolescents with OW and OB also consumed higher levels of added sugar, saturated fat, and sodium from snacks than adolescents with NW. By categorizing adolescents according to three weight classifications, normal weight, overweight and obesity, the findings suggest a potential dose response between snacking frequency and mean snack size and the association with weight status. Trends were consistent when examining associations between snacks from foods only and beverages only (Supplementary Materials, Table S1–S4, available online at www.mdpi.com/xxx/s1). These findings, while cross-sectional, provide population representative evidence of snacking associations with weight outcomes and suggest that approaches to obesity prevention that target snacking are relevant for adolescents.

Results are consistent with other studies that have used similar approaches to examine snacking frequency and weight status in children [19,20]. In a study by Murakami and Livingstone, snacking frequency was associated with higher risks of overweight and abdominal obesity in adolescents, after controlling for EI/EER. In a study examining associations between snacking frequency and weight status in younger children aged 1 to 5 years, positive associations were observed when snacking definitions took into account other eating occasions between meals (in addition to eating occasions identified as snacks) [20]. Based on this previous work, the current study expanded the definition of snacking to include self-identified snacking occasions plus other eating occasions between meals, and accounted for reporting bias in the analyses.

This study extends the current literature by examining snack size as an important dimension of snacking and the findings suggest that in addition to frequency, snack size seems to be important as it relates to obesity risk in adolescents. Taking into account the frequency and energy density of snacks consumed, adolescents at healthier weights are consuming smaller snacks. More work is needed to identify the optimal number and size of snacks for appropriate growth. Current dietary recommendations for adolescents do not clearly specify the number, size or type of snacks that should be consumed to promote optimal nutrient intake and manage weight.

Interestingly, the findings from this study do not provide evidence that adolescents with OW and OB are consuming more energy dense snacks compared to adolescents with NW, which is contrary to much of the existing literature on this topic [7,38]. After controlling for snack frequency and size, adolescents with OB consumed snacks that had a lower mean energy density per snack than children with NW. Energy density is defined as the number of calories per gram of food and can range from zero to nine, based on the composition of water and fat in the food (water has an energy density of zero kcal/g and fat has an energy density of 9 kcal/g) [39]. Energy density has been examined as a predictor of diet quality in children (e.g., lower energy density is associated with higher diet quality) and increasing intake of lower energy dense foods has been suggested as a strategy for weight management [40,41].

While energy density can act as a proxy for quality relevant to obesity, it does not fully capture nutritional quality or types of foods. In this study, mean snack energy density did not differ significantly between adolescents with NW and those with OW, and the difference between adolescents with NW and adolescents with OB was modest. On average, snack energy density in the total sample was 2.6 kcal/g/occasion, which is considered medium energy density and includes food items like breads, meats, cheeses, pretzels and popcorn [41]. Energy density in this range could reflect the consumption of these foods or of a combination of lower energy density foods (like fruits and vegetables) and nutrient-rich energy dense foods (like nuts and seeds). Since it is not possible to discern the types of foods from this study, further analyses are needed in order to characterize the types, sources and quality of snacks foods that adolescents are consuming.

This study contributes new evidence to the literature regarding snack intake and weight status in adolescents and has a number of strengths. First, three dimensions of snacking were examined including mean daily snacking occasions, mean snack size (kcal/occasion), and mean snack energy density (kcal/g/occasion). To our knowledge, this is the first study to include multiple snacking parameters in analyses examining the associations of snacking with weight status in adolescents. Multiple-pass 24-h dietary recalls were used to assess dietary intake and adolescents self-reported their intake [42]. While even this gold standard method is susceptible to bias, this study accounted for energy misreporting and evidence suggests that any misreporting most likely underestimated actual intake, especially in adolescents with higher weight status [43]. Findings from this study show that snacking varies by weight status, but also by gender and race/ethnicity. Additional studies are needed to examine context-specific factors that might differentially impact snacking behaviors these groups. For example, targeted marketing of snack foods might be influencing higher consumption among black adolescents and a deeper understanding could yield specific targets to improve dietary intake and address diet-related health disparities [44].

NHANES provides population-representative data in a large sample of US adolescents, but it is important to note limitations due to the cross-sectional design. Longitudinal research needs to be conducted to further elucidate the relationships found in this study. For example, snack frequency and snack size could be contributing to excess caloric intake and subsequent adiposity, but it is also possible that adolescents with overweight or obesity may have characteristics, such as impulsive eating, that make them more susceptible to snacking [45]. Further, while various approaches have been used to address measurement challenges, dietary measures are highly susceptible to reporting bias and the approach taken may not be sufficient to address those sources of measurement error. Another measurement limitation is the lack of a robust physical activity measure to account for energy expenditure. Exercise has been linked with compensatory snacking behavior, particularly unhealthy snacks, and this should be an area of future research, especially for growing children [46]. Lastly, the snacking measures from this study do not take into consideration the social, behavioral or environmental factors that may influence snack choices and these factors are important for developing salient targets for adolescents [47].

## 5. Conclusions

The nationally representative findings in this study provide evidence that US adolescents with OW and OB consume more snacks daily and more calories at each snacking occasion compared to adolescents with NW. Adolescents with OW and OB also consume greater amounts of added sugar, saturated fat and sodium from snacks than adolescents with NW, but consume snacks with a lower average energy density than NW adolescents. These findings suggest that snacking behaviors may be an important, multi-dimensional target for population-based approaches to reducing obesity risk among adolescents. Specifically, results suggest that the size of snacks consumed by adolescents as well as the frequency of daily snacking may have implications for weight status. Additional research is needed to determine the prospective association between snacking and weight status in adolescents. Future studies should also better characterize the types of foods that adolescents consume as snacks and how snacking behavior impacts overall diet quality. This information is critical for developing appropriate evidenced-based dietary recommendations for this age group and identifying effective obesity prevention and treatment targets.

## Figures and Tables

**Table 1:** Demographic characteristics (*n* = 6545).

	*n*	%
Child		
Age	15.4 (0.05)	
Race		
Non-Hispanic White	1798	57.7
Mexican American/Hispanic	2281	20.3
Non-Hispanic Black	1799	14.7
Other	671	7.3
Gender		
Male	3287	49.7
Female	3262	50.3
Weight status ^1^		
Normal weight	4062	64.6
Overweight	1097	15.3
Obese	1390	20.1
BMI	23.9 (0.14) kg/m^2^	
BMI z-score	0.57 (0.03)	
Number of Daily Snacking Occasions	1.77 (0.02)	
Snack size (kcal per occasion)	284.54 (4.60)	
Snack energy density (kcal/g per occasion ^2^	2.56 (0.02)	
Caregiver		
Age	43.9 (0.36)	
Ratio of income to poverty ^3^	2.6 (0.06)	
Low	2417	28.5
High	4132	71.5
Education ^4^		
High school or less	3229	41.8
More than high school	3065	58.2
Marital status ^5^		
Partnered	4150	72.6
Not partnered	2066	27.4

^1^ BMI-for-age percentiles: normal weight <85th; overweight ≥85th to <95th; obese ≥95th [36,37]; ^2^ *n* = 6563. ^3^ Calculated by dividing family income by the Health and Human Services’ poverty guideline, specific to family size, as well as the year and state [26]; *n* = 6088; ^4^ *n* = 6294; ^5^ *n* = 6216.

**Table 2:** Snack parameters (food and beverages) by adolescent demographic characteristics (*n* = 6545) ^1^.

	Number of Daily Snacking Occasions ^2^			Snack Size (kcal/occasion) ^3^			Snack Energy Density (kcal/g/occasion) ^4^		
	Mean	SE	*p*	Mean	SE	*p*	Mean	SE	*p*
Weight status ^5^									
Normal ^6^	1.69	0.02	-	262.32	4.41	-	2.62	0.03	-
Overweight	1.85	0.05	0.005	305.41	8.84	<0.001	2.51	0.06	0.09
Obese	1.97	0.05	<0.001	339.60	10.12	<0.001	2.41	0.06	0.005
Gender									
Male	1.96	0.03	-	342.39	7.60	-	2.56	0.03	-
Female	1.59	0.03	<0.001	227.28	4.67	<0.001	2.57	0.04	0.79
Race									
White	1.75	0.03	-	285.66	6.08	-	2.54	0.04	-
Mexican American/ Hispanic	1.76	0.03	0.82	270.33	6.30	0.07	2.54	0.03	0.94
Black	1.91	0.04	0.001	319.78	6.11	<0.001	2.7	0.04	0.001
Other	1.77	0.06	0.77	243.72	9.3	<0.001	2.54	0.07	0.97
Poverty-income-ratio									
Low	1.76	0.03	-	291.21	6.75	-	2.53	0.03	-
High	1.78	0.02	0.69	281.79	4.83	0.23	2.57	0.03	0.35

^1^ Adjusted for dietary weights, survey cycle year, child age, HH age, EI/EER, and mean meal size. ^2^ Adjusted for mean snack size and energy density of snacks. ^3^ Adjusted for snacking frequency and energy density of snacks. ^4^ Adjusted for mean snack size and snacking frequency. ^5^ BMI-for-age percentiles: normal weight <85th; overweight ≥85th to <95th; obese ≥95th percentile. ^6^ *n* = 4062, 48.9% female, 15.5(0.7) years; 61.3% Non-Hispanic White, 17.8% Mexican American/Hispanic, 13.4% Non-Hispanic Black, 7.5% other race. Kcal: kilocalories; g: gram; SE: standard error, HH: head of household; EI/EER: ratio of reported energy intake to estimated energy requirement; - not applicable.

**Table 3:** Mean daily intake of added sugar, saturated fat, and sodium from snacks by adolescent demographic characteristics (*n* = 6545).

	Added Sugar (kcal) ^1^			Saturated Fat (kcal) ^1^			Sodium (mg) ^1^		
	Mean	SE	*p*	Mean	SE	*p*	Mean	SE	*p*
Weight status ^2^									
Normal ^3^	120.52	3.78	-	48.42	1.40	-	456.04	14.10	-
Overweight	158.99	8.12	<0.001	61.79	2.65	<0.001	555.12	25.02	<0.002
Obese	183.54	9.50	<0.001	72.38	4.31	<0.001	677.37	39.93	<0.001
Gender									
Male	175.68	6.90	-	69.69	2.52	-	668.28	26.04	-
Female	102.96	3.52	<0.001	41.07	1.55	<0.001	365.15	15.42	<0.001
Race									
White	148.14	6.07	-	54.77	2.00	-	497.62	15.94	-
Mexican American/Hispanic	110.69	3.88	<0.001	51.25	1.76	0.20	498.54	16.55	0.97
Black	154.9	4.76	0.37	65.82	2.31	<0.001	618.6	23.83	<0.001
Other	115.09	10.11	<0.005	49.49	2.78	0.15	500.44	39.23	0.95
Poverty-income-ratio									
Low	152.61	5.95	-	55.69	1.96	-	524.15	20.25	-
High	133.7	4.26	<0.003	55.13	1.41	0.78	512.41	12.97	0.6

^1^ Adjusted for dietary weights, survey cycle year, child age, HH age, EI/EER, and mean meal size. ^2^ BMI-for-age percentiles: normal weight <85th; overweight ≥85th to <95th; obese ≥95th. ^3^ *n* = 4,062, 48.9% female, 15.5 (0.7) years; 61.3% Non-Hispanic White, 17.8% Mexican American/Hispanic, 13.4% Non-Hispanic Black; 7.5% other race. Kcal: kilocalories; g: gram; SE: standard error, HH: head of household; EI/EER: ratio of reported energy intake to estimated energy requirement; - not applicable.

## Supplementary Materials

The following are available online at https://www.mdpi.com/2072-6643/11/7/1486/s1, Table S1: Food-only snacking parameters by adolescent demographic characteristics (*n* = 6223), Table S2: Beverage-only snacking parameters by adolescent demographic characteristics (*n* = 4736), Table S3: Total daily snacking contributions from added sugar, saturated fat, and sodium from food only, by adolescent demographic characteristics (*n* = 6464), Table S4: Total daily snacking contributions from added sugar, saturated fat, and sodium in beverages only, by adolescent demographic characteristics (*n* = 5615).
