# Supplementary material for: Associations between Snacking and Weight Status among Adolescents 12–19 Years in the United States
Source: Nutrients. 2019 Jun 29;11(7):1486. doi: 10.3390/nu11071486 (PMC6682988; doi:10.3390/nu11071486)
Supplement: Supplementary file 1 [file nutrients-11-01486-s001.pdf]

nutrients

Table S1. Food-only snacking parameters by adolescent demographic characteristics (n=6223)[1].

	Number of daily snacking occasions[2]			Snack size (kcal/occasion)[3]			Snack energy density (kcal/g/occasion)[4]		
	**Mean**	**SE**	**p**	**Mean**	**SE**	p	Mean	SE	p
Weight status[5]									
Normal	1.97	0.03	-	184.78	3.61	-	3.4	0.04	-
Overweight	2.11	0.06	0.04	213.01	9.17	0.003	3.48	0.05	0.25
Obese	2.26	0.07	<0.001	253.68	17.14	<0.001	3.35	0.06	0.48
Gender									
Male	2.328	0.04	-	238.64	10.13	-	3.44	0.03	-
Female	1.82	0.04	<0.001	167.35	4.41	<0.001	3.36	0.04	0.16
Race									
White	1.96	0.04	-	204.38	6.59	-	3.41	0.04	-
Mexican American/ Hispanic	2.12	0.04	0.008	186.7	5.97	0.04	3.31	0.04	0.08
Black	2.29	0.05	<0.001	230.6	8.26	<0.001	3.53	0.05	0.04
Other	2.09	0.08	0.19	174.17	6.82	<0.001	3.28	0.07	0.08
Poverty-income-ratio									
Low	2.04	0.05	-	195.66	5.23	-	3.42	0.04	-
High	2.05	0.03	0.9	205.21	6.89	0.27	3.39	0.03	0.59

[1] Adjusted for dietary weights, survey cycle year, child age, HH age, EI/EER, and mean meal size; [2] Adjusted for mean snack size, and energy density of snacks; [3] Adjusted for snacking frequency, and energy density of snacks; [4] Adjusted for mean snack size, and snacking frequency; [5] BMI-for-age percentiles: normal weight <85th; overweight ≥85-<95th; obese ≥ 95th; Kcal: kilocalories; g: gram; SE: standard error, HH: head of household; EI/EER: ratio of reported energy intake to estimated energy requirement; - not applicable.

Table S2. Beverage-only snacking parameters by adolescent demographic characteristics (n=4736)[1].

	Number of daily snacking occasions[2]			Snack size (kcal/occasion)[3]			Snack energy density (kcal/g/occasion)[4]		
	**Mean**	**SE**	**p**	**Mean**	**SE**	**p**	**Mean**	**SE**	**p**
Weight status[5]									
Normal	0.98	0.02	-	163.68	5.2	-	0.55	0.02	-
Overweight	1.13	0.04	0.007	176.35	6.38	0.12	0.54	0.02	0.73
Obese	1.17	0.04	<0.001	205.51	12.45	0.006	0.5	0.02	0.1
Gender									
Male	1.15	0.02	-	205.11	9.02	-	0.53	0.02	-
Female	0.93	0.02	<0.001	141.53	4.93	<0.001	0.55	0.02	0.54
Race									
White	1.08	0.02	-	187.22	6.28	-	0.54	0.02	-
Mexican American/ Hispanic	0.99	0.03	0.009	149.94	4.48	<0.001	0.56	0.02	0.41
Black	1	0.02	0.012	162.13	4.91	0.001	0.5	0.01	0.22
Other	0.99	0.04	0.07	160.68	9.25	0.012	0.62	0.04	0.07
Poverty-income-ratio									
Low	1.07	0.02	-	185.09	8.35	-	0.51	0.02	-
High	1.03	0.02	0.19	169.39	4.29	0.09	0.55	0.02	0.07

[1] Adjusted for dietary weights, survey cycle year, child age, HH age, EI/EER, and mean meal size; [2] Adjusted for mean snack size, and energy density of snacks; [3] Adjusted for snacking frequency, and energy density of snacks; [4] Adjusted for mean snack size, and snacking frequency; [5] BMI-for-age percentiles: normal weight <85th; overweight ≥85-<95th; obese ≥ 95th; Kcal: kilocalories; g: gram; SE: standard error, HH: head of household; EI/EER: ratio of reported energy intake to estimated energy requirement; - not applicable.

Table S3. Total daily snacking contributions from added sugar, saturated fat, and sodium from food only, by adolescent demographic characteristics (n=6464).

	**Added sugar (kcal)[1]**			**Saturated fat (kcal)[1]**			**Sodium (mg)[1]**		
	**Mean**	**SE**	**p**	**Mean**	**SE**	p	Mean	SE	p
Weight status[2]									
Normal	60.97	1.76	-	46.13	1.42	-	432.65	13.91	-
Overweight	71.47	3.35	0.004	58.6	2.95	<.001	519.97	28.67	0.01
Obese	86.54	5.36	<0.001	71.47	4.47	<.001	670.79	47.94	<0.001
Gender									
Male	80.94	3.08	-	67.11	2.87	-	635.16	29.94	-
Female	54.67	2.16	<0.001	39.35	1.51	<.001	354.54	16.29	<0.001
Race									
White	65.62	2.36	-	52.8	2.15	-	471.78	19.61	-
Mexican American/ Hispanic	59.85	2.98	0.16	46.98	1.82	0.04	471.46	18.45	0.99
Black	89.62	3.16	<0.001	65.94	2.41	<.001	611.14	24.9	<0.001
Other	61.32	7.66	0.56	46.54	4.1	0.2	488.83	48.77	0.75
Poverty-income-ratio									
Low	67.24	2.65	-	53.51	1.9	-	494.96	20.84	-
High	67.86	2.06	0.85	52.94	1.64	0.79	493.05	17.48	0.94

[1] Adjusted for dietary weights, survey cycle year, child age, HH age, EI/EER, and mean meal size; [2] BMI-for-age percentiles: normal weight <85th; overweight ≥85-<95th; obese ≥ 95th; Kcal: kilocalories; g: gram; SE: standard error, HH: head of household; EI/EER: ratio of reported energy intake to estimated energy requirement; - not applicable.

Table S4. Total daily snacking contributions from added sugar, saturated fat, and sodium from beverages only, by adolescent demographic characteristics (n=5615).

	**Added sugar (kcal)[1]**			**Saturated fat (kcal)[1]**			**Sodium (mg)[1]**		
	**Mean**	**SE**	**p**	**Mean**	**SE**	p	Mean	SE	p
Weight status[2]									
Normal	95.94	4.14	-	9.12	0.65	-	82.05	4.27	-
Overweight	125.57	10.66	0.01	9.76	1.15	0.6	96.83	8.91	0.13
Obese	141	9.71	<0.001	12.98	3.4	0.26	110.54	9.31	0.006
Gender									
Male	138.24	7.53	-	11.53	1.17	-	115.39	6.9	-
Female	80.05	4.05	<0.001	8.44	0.95	0.008	63.82	4.25	<0.001
Race									
White	124.58	6.95	-	10.85	1.37	-	102.23	5.96	-
Mexican American/ Hispanic	79.51	3.9	<0.001	10	0.83	0.55	78.43	4.99	0.002
Black	105.11	4.95	0.02	6.75	0.67	0.003	65.07	4.49	<0.001
Other	88.54	8.81	0.001	10.41	1.67	0.83	81.31	10.89	0.09
Poverty-income-ratio									
Low	127.43	6.87	-	8.54	0.83	-	91.98	5.99	-
High	102.71	4.81	0.001	10.63	1.09	0.04	89.56	4.42	0.71

[1] Adjusted for dietary weights, survey cycle year, child age, HH age, EI/EER, and mean meal size; [2] BMI-for-age percentiles: normal weight <85th; overweight ≥85-<95th; obese ≥ 95th; Kcal: kilocalories; g: gram; SE: standard error, HH: head of household; EI/EER: ratio of reported energy intake to estimated energy requirement; - not applicable.
